# *Salmonella* Typhi Vertebral Osteomyelitis and Epidural Abscess

**DOI:** 10.1155/2016/6798157

**Published:** 2016-03-10

**Authors:** Hau Wei Khoo, Ying Ying Chua, John L. T. Chen

**Affiliations:** ^1^Department of Orthopaedics Surgery, Singapore General Hospital, Outram Road, Singapore 169608; ^2^Department of Infectious Diseases, Singapore General Hospital, Outram Road, Singapore 169608

## Abstract

*Salmonella* vertebral osteomyelitis is an uncommon complication of* Salmonella* infection. We report a case of a 57-year-old transgender male who presented with lower back pain for a period of one month following a fall. Physical examination only revealed tenderness over the lower back with no neurological deficits. MRI of the thoracic and lumbar spine revealed a spondylodiscitis at T10-T11 and T12-L1 and right posterior epidural collection at the T9-T10 level. He underwent decompression laminectomy with segmental instrumentation and fusion of T8 to L3 vertebrae. Intraoperatively, he was found to have acute-on-chronic osteomyelitis in T10 and T11, epidural abscess, and discitis in T12-L1. Tissue and wound culture grew* Salmonella* Typhi and with antibiotics susceptibility guidance he was treated with intravenous ceftriaxone for a period of six weeks. He recovered well with no neurological deficits.

## 1. Introduction

*Salmonellae* are known to cause a broad spectrum of human illnesses from gastroenteritis, typhoid fever, and bacteraemia to the asymptomatic carrier state [1]. An uncommon complication of* Salmonella* infection is vertebral osteomyelitis. While combination of vertebral osteomyelitis and epidural abscess is rare, accounting for only 1–4% of all bone infections, typhoid osteomyelitis of the spine has been reported to be extremely rare [2, 3]. In this paper, we describe a rare case of a 57-year-old transgender male with* Salmonella* Typhi vertebral osteomyelitis and epidural abscess.

## 2. Case Report

A 57-year-old transgender male with no past medical history, presented with lower back pain following a mechanical fall when he tripped and fell on his back a month ago. He sustained a minor scalp laceration. Otherwise, there was no other injury noted. He sought medical attention as he had worsening lower back pain. There were no symptoms of radiculopathy or urinary or bowel incontinence. He denied any fever, constitutional symptoms, ill contact, or recent travel. Systemic review was unremarkable. Physical examination was largely normal with no neurological deficits noted. There was spinal tenderness noted over the lower thoracic and lumbar area. Digital rectal examination revealed an intact anal tone with no saddle anaesthesia. His total white count was mildly elevated at 11.54 × 10^9^/L and ESR and CRP were elevated at 106 mm/Hr and 33.4 mg/L, respectively. HIV screen was negative.

**Figure 1:**
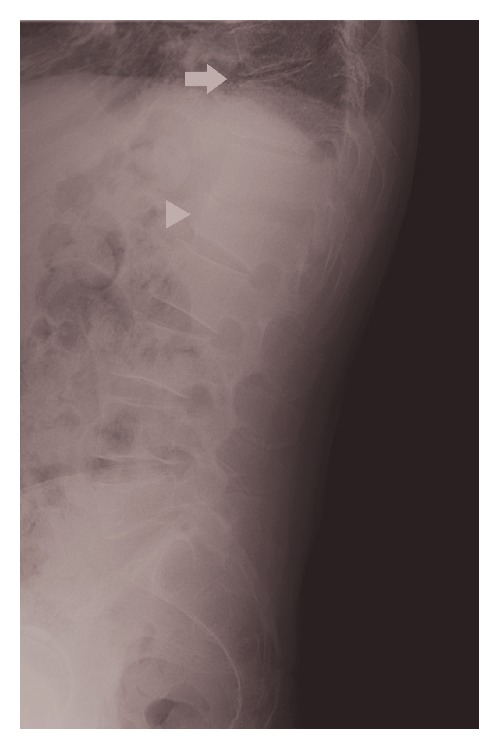
Plain radiograph of the lumbar spine revealed severe osteoarthritic changes of the thoracic and lumbar spine. There were compression collapse of T10/T11 vertebrae with mild retrolisthesis (white arrow) and a grade-one-to-two compression collapse of L1 vertebra with minimal retrolisthesis (white arrowhead).

**Figure 2:**
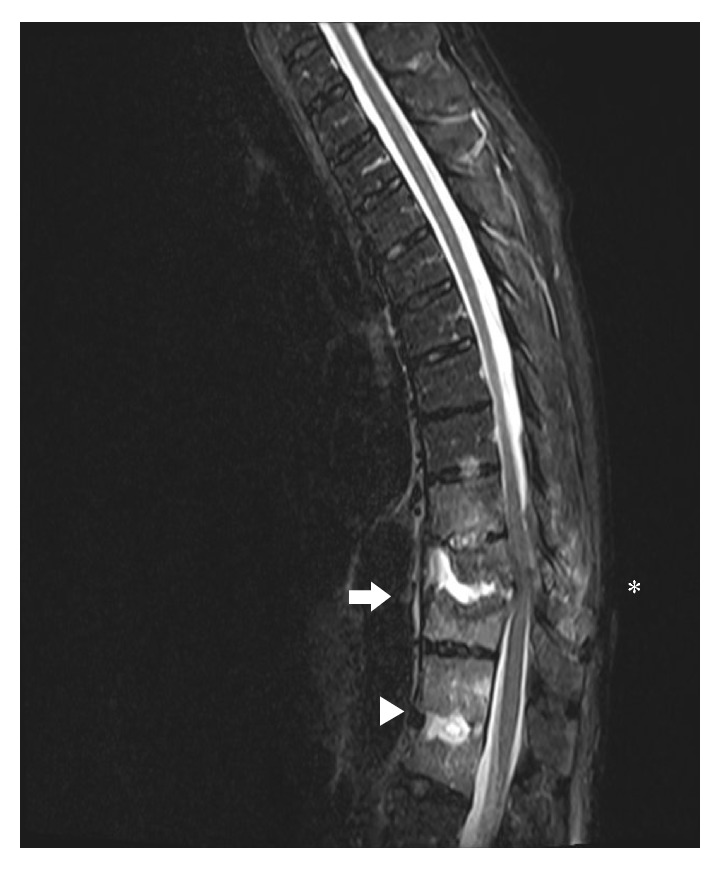
MRI of the thoracic and lumbar spine revealed spondylodiscitis in the thoracolumbar spine, worst at the T10-T11 level (white arrow) where there is associated paravertebral and epidural components with compression of the spinal cord. There is also oedema seen in the spinous processes of T9-T10 and the adjacent paravertebral muscles (Asterisk). Spondylodiscitis was also seen at T12/L1 level (white arrowhead).

Plain X-ray of the thoracic and lumbar spine revealed compression fracture of T10/T11 vertebrae with mild retrolisthesis and grade-one-to-two compression collapse of L1 vertebra with minimal retrolisthesis (Figure 1). MRI thoracic and lumbar spine showed spondylodiscitis at T10-T11 and T12-L1 where there were associated paravertebral and epidural components with compression of the spinal cord (Figure 2).

**Figure 3:**
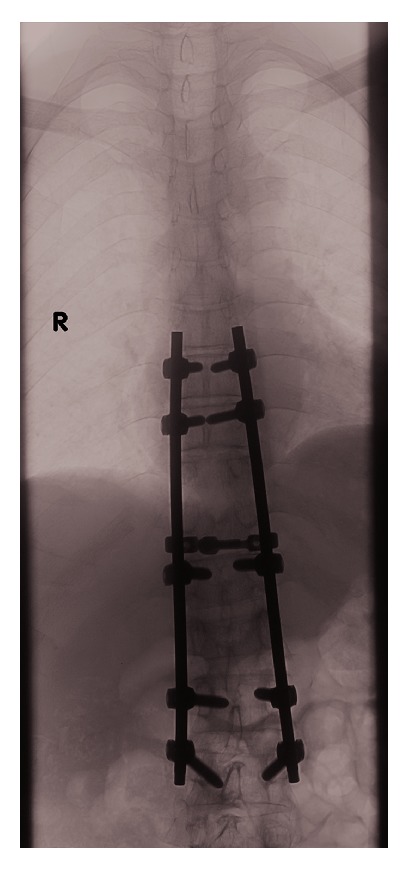
Postoperative plain radiograph showing segmental instrumentation from T8 to L3 vertebrae.

**Figure 4:**
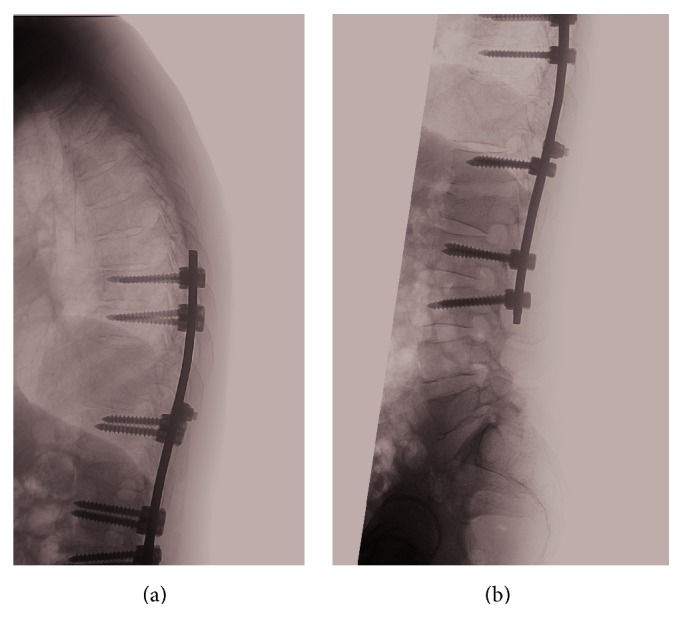
Postoperative plain radiograph showing segmental instrumentation from T8 to L3 vertebrae.

He underwent decompression laminectomy and left costotransversectomy, T10-T11, with T8 to L3 segmental instrumentation and posterior fusion with fluoroscopic navigation. Intraoperatively, he was found to have acute-on-chronic osteomyelitis in T10 and T11, epidural abscess, and discitis in T12-L1. Postoperative radiograph is shown in Figures 3 and 4. Postoperatively, he recovered well with no neurological deficits. Tissue and wound culture from surgical specimens grew* Salmonella* Typhi sensitive to ceftriaxone. He was given intravenous ceftriaxone for a total of 6 weeks with outpatient antibiotics therapy. He had follow-up with orthopaedics spine surgeon and infectious disease physician with good recovery.

## 3. Discussion

*Salmonella* has been recognised as a causative organism of osteomyelitis for more than a century [4].* Salmonella* osteomyelitis can be divided into two broad categories: typhoid osteomyelitis (*Salmonella* Typhi and paratyphi) and non-typhoid osteomyelitis. While* Salmonella* osteomyelitis is uncommon, typhoid osteomyelitis of the spine is reported as extremely rare [3].

Review by Santos and Sapico revealed that 54% with* Salmonella* osteomyelitis had predisposing conditions for infection while the remaining 46% did not have any predisposition [4]. Osteomyelitis typically occurs in patient with sickle cell disease and those who are immunocompromised; however, it is a rare cause of osteomyelitis in patients with no sickle cell disease, accounting for approximately 0.5% of all cases [5, 6]. Other common underlying conditions are atherosclerosis, diabetes, collagen diseases, liver cirrhosis, and achlorhydria. Our patient was not found to have any of those predisposing factors. Common presenting symptoms were fever and back and neck pain, which are universal initial complaints in* Salmonella* vertebral osteomyelitis and pyogenic vertebral osteomyelitis [4]. However, pain experienced in vertebral osteomyelitis offers no diagnostic characteristics [3]. Therefore, patients presenting with back pain associated with febrile illness should be fully investigated with imaging studies to rule out vertebral osteomyelitis. However, in this case, the patient presented atypically with only back pain but afebrile. Therefore, there should be a high index of suspicion in ruling out osteomyelitis as a cause of the back pain. Most frequent site of infection was the lumbar vertebrae (50%) followed by thoracic vertebrae (20%) and the rest involved multiple bones and joints [4].

In assessment of spinal infection, MRI is the most sensitive, specific, and accurate imaging tool [7]. Nevertheless, definitive diagnosis of* Salmonella* vertebral osteomyelitis depends on the isolation of the organism from a bone specimen obtained by needle or open biopsy, aspirate of an adjacent fluid collection, or positive blood culture in a patient with clinical features consistent with vertebral osteomyelitis [4].

*Salmonella* vertebral osteomyelitis can be primarily treated with antibiotics therapy and should be guided by results of antibiotics susceptibility studies for a duration of at least 6 weeks. Chloramphenicol, tetracycline, ampicillin, trimethoprim-sulfamethoxazole, amikacin, and cefotaxime have been used for treatment of* Salmonella* vertebral osteomyelitis [4]. However, of late, there has been emergence of antibiotics resistance mainly in South East Asia, the Middle East, and Africa and therefore agents such as third-generation cephalosporins and fluoroquinolones are the agents of choice [2]. Patients with large paravertebral abscesses may need only needle aspiration while patients with extensive bone destruction and epidural abscesses will require operative procedures such as laminectomy with abscess drainage, debridement and curettage, anterior debridement, and fusion with autologous bone grafting [4].
